# Synthetic toehold biosensors for detecting *Listeria monocytogenes* in raw milk

**DOI:** 10.3389/fmicb.2026.1891217

**Published:** 2026-07-24

**Authors:** Víctor M. Carballo-Uicab, Luz E. Casados-Vázquez, Drew Endy, José E. Barboza-Corona

**Affiliations:** 1 Graduate Program in Biosciences, Life Science Division, University of Guanajuato Campus Irapuato-Salamanca, Guanajuato, Mexico; 2 Department of Food, Life Science Division, University of Guanajuato Campus Irapuato-Salamanca, Guanajuato, Mexico; 3 SECIHTI-University of Guanajuato, Guanajuato, Mexico; 4 Department of Bioengineering, Stanford University, Stanford, CA, United States

**Keywords:** Cell-free biosensing, *Listeria*, RNA biosensors, synthetic biology, pathogen detection

## Abstract

**Introduction:**

To our knowledge, direct detection of foodborne bacterial pathogens using toehold biosensors has not been demonstrated in complex food matrices such as raw milk, particularly for *Listeriamonocytogenes*. Here, we report the design and evaluation of toehold biosensors for detecting synthetic triggers and *L. monocytogenes* in both pure cultures and raw milk.

**Methods:**

We performed a phylogenetic analysis of the V2-V3 hypervariable regions of the 16S rRNA gene, showing that *Listeria* spp. rRNA are distinct from other bacterial groups; a 36-nt trigger in the V2 region matched *Listeria* spp., whereas non-*Listeria* bacteria exhibited multiple mismatches. We designed several toehold sequences specific to the V2 region of *L. monocytogenes* and constructed three (A, B, C), selecting toehold B because of its highest experimental stability.

**Results:**

Without NASBA amplification, toehold B responded to synthetic RNA triggers, inputs ranging from 10 to 10^12^ copies (50 aM to 5 μM); these were used to evaluate sensor activation and to select a working RNA concentration for subsequent assays with biological RNA. Maximum activation was observed at 10^12^ and 10^11^ RNA copies, corresponding to 5 μM and 500 nM, respectively, yielding 17- and 5-fold changes at 30 min. A TRIzol-based protocol was used to extract bacterial RNA from pure culture or milk, producing consistent results in cell-free assays.*Listeria* RNA extracted with either a column-based kit or TRIzol reached a maximum absorbance of 3.5 at 600 nm. RNA from pure bacterial cultures of *Listeria* exhibited a fold change of 1.8, compared to approximately 1.5 when the toehold was activated with RNA obtained from crude milk contaminated with *Listeria*, confirming that the toehold biosensor can detect *L. monocytogenes*-derived RNA in a complex milk matrix. RNA from uncontaminated crude milk showed an activation ratio of 0.73, which differed statistically from the previous values.

**Conclusion:**

Although the activation ratios are low, this first proof-of-concept demonstration shows that toehold-switch biosensors are activated by *L. monocytogenes* RNA from a complex food matrix (raw milk), not only by short synthetic triggers, suggesting a promising path toward more prevalent and affordable food security diagnostics. This finding remains a meaningful advance because detection is achieved in a cell-free format without nucleic acid amplification.

## 1 Introduction

The World Health Organization has reported 600 million cases worldwide from contaminated food and 420,000 deaths annually (World Health Organization, 2026). Foodborne pathogens cause numerous diseases with profound impacts on human health (Bintsis, 2017). For example, *Listeria monocytogenes* causes a serious foodborne illness, especially in vulnerable groups such as children, pregnant women, and the elderly, and is considered pathogenic to humans (Osek and Wieczorek, 2022; Orsi et al., 2023). Although *L. innocua* is generally considered nonpathogenic, some strains can cause disease in humans (Johnson et al., 2004; Perrin et al., 2003). Both bacteria, *L. monocytogenes* and *L. innocua* are the most common *Listeria* species found in food, and they serve as indicators of food safety (Barboza-Corona et al., 2026). Foodborne pathogen outbreaks cause significant losses and strain healthcare and economic systems, underscoring the need to develop rapid diagnostic methods for pathogen detection (Chen et al., 2025; Koksaldi et al., 2024). Common methods for detecting foodborne pathogens include microbiological testing, biochemical identification, PCR-based methods, and loop-mediated isothermal amplification (LAMP) (Barboza-Corona et al., 2026; Haddad et al., 2025; Aladhadh, 2023). Standard microbiological procedures are highly reliable but may require 3 to 5 days to complete when enrichment, isolation, and confirmation steps are included. Molecular and isothermal amplification methods, including PCR and LAMP, considerably shorten analytical time; for example, LAMP assays for *Listeria* spp. in raw milk have been reported to operate within approximately 1.5 h (Barboza-Corona et al., 2026). However, raw milk remains a challenging matrix for nucleic-acid-based detection because fats, proteins, calcium, somatic cells, endogenous microbiota, and other matrix-associated components may affect nucleic acid recovery, enzymatic reactions, or optical readouts (Moon et al., 2022; Schrader et al., 2012). Therefore, regardless of the downstream detection chemistry, matrix-compatible sample preparation is essential. In this context, amplification-free cell-free biosensors may offer a complementary strategy for rapid screening when paired with optimized RNA extraction from raw milk.

In this regard, developing methods that demonstrate high sensitivity, specificity, speed, stability, and portability, and that do not require specialist training to operate, is necessary (Barboza-Corona et al., 2026; Stark et al., 2018; Park et al., 2013) Biosensors generated through engineered biology are a promising option, as they have demonstrated speed and specificity in detecting target analytes and have applications in food safety, medical diagnostics, biotechnology, and the pharmaceutical industry (Priya, 2024; Khan et al., 2022). Synthetic biology has enabled the development of programmable biosensors that recognize molecular inputs and produce measurable outputs. Whole-cell, cell-free, and RNA-based biosensors are emerging tools for molecular detection and diagnostics (Liu et al., 2024; Hameed et al., 2018). Among RNA-based platforms, toehold switch biosensors are particularly attractive because they can be computationally designed, are orthogonal, and detect specific RNA sequences that trigger the expression of reporter proteins in cell-free systems (Takahashi et al., 2018; Green et al., 2014).

**Figure 1:**
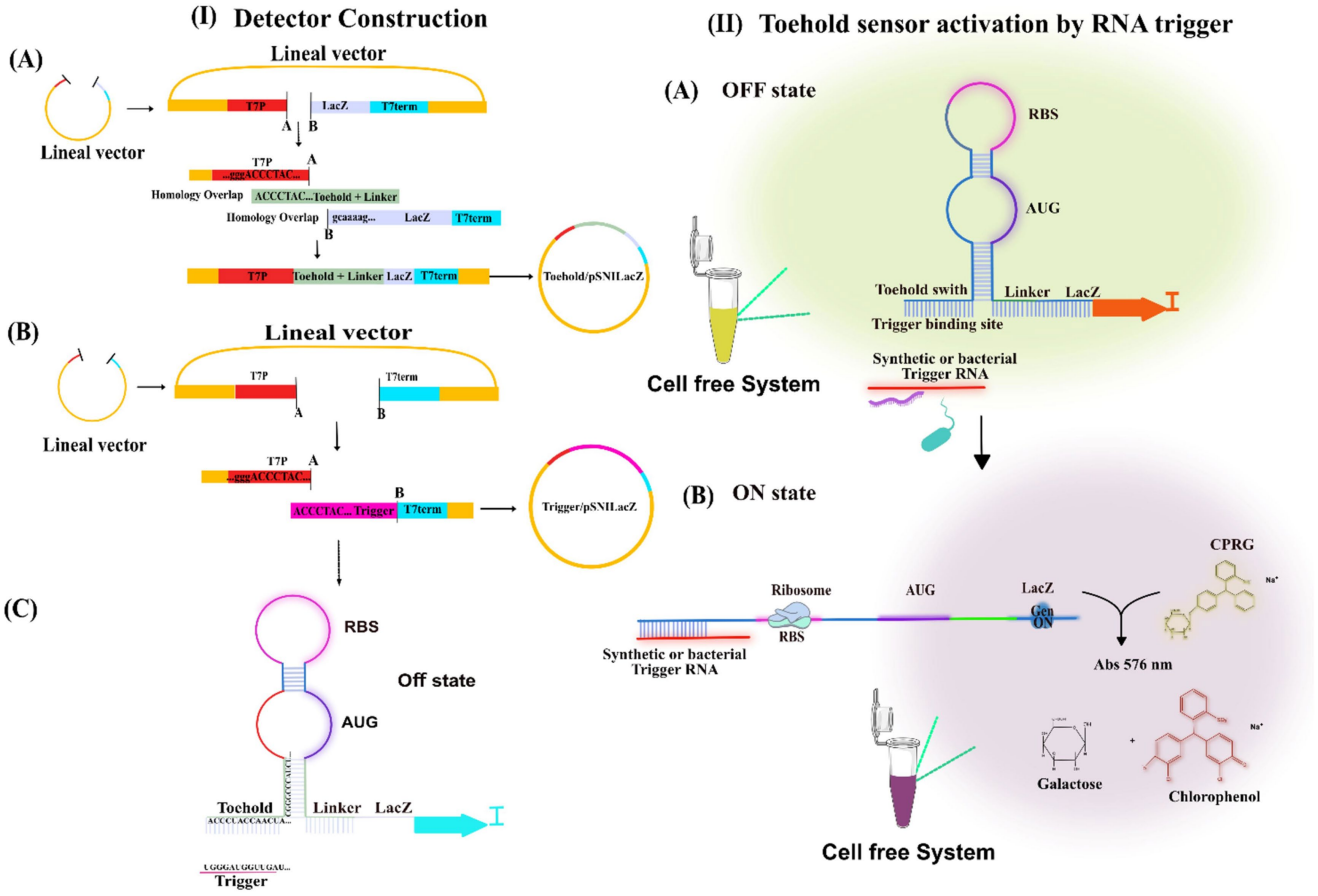
Biosensor construction and activation. **(I)** Construction. (**A,B**) Synthetic toehold and trigger were assembled into the pSNI_plasmid_sensor_LacZ using Gibson assembly. Both were placed under the control of the pT7 promoter and pT7 terminator. The toehold switch was positioned downstream of the *lacZ* gene. (**C**) During transcription, a switch RNA forms a loop structure that sequesters a ribosome-binding site (RBS) and contains a toehold designed to complement the trigger RNA sequence. **(II)** Toehold switch sensor activation by RNA trigger. **(A)** Triggers can be synthetic, derived from a pure bacterial culture, or from food suspected of bacterial contamination (e.g., crude milk). A trigger is added to a cell-free reaction containing the toehold sensor. **(B)** The toehold switch specifically binds to the target RNA; the stem-loop structure opens; and the ribosomes (RBS) gain access to the ribosome binding site, starting translation and producing *β*-galactosidase (i.e., *lacZ*). This enzyme then cleaves the substrate chlorophenol red-β-galactopyranoside (CPRG), causing a color shift from yellow to purple. Figure created with Affinity Designer 2.6.4.

Alternatively, RNA toehold switches serve as powerful tools for detecting almost any RNA sequence. A toehold switch contains a complementary sequence to a target sequence, followed by a hairpin structure with a hidden ribosome binding site (RBS) that prevents ribosomal recognition, thereby inhibiting the translation of the reporter gene. When a trigger RNA of the target microorganisms binds to the toehold switch sequence, the hairpin unfolds, exposing the RBS to enable ribosomal recognition and protein synthesis. Then, the reporter protein can generate a visible fluorometric signal or, upon addition of a substrate, a colorimetric signal (Diaz et al., 2025; Koksaldi et al., 2024; Ekdahl et al., 2022; Takahashi et al., 2018; Green et al., 2014; Pardee et al., 2014) (Figure 1). Toehold biosensors have been used to detect viruses (Ebola, Zika, and SARS-CoV-2) (Chen et al., 2025; Chakravarthy et al., 2021; Park and Lee, 2021; Pardee et al., 2016, 2014), bacteria from the human gut microbiota, and endogenous *E. coli* small RNA (Sarkar et al., 2025; Takahashi et al., 2018; Green et al., 2014).

To the best of our knowledge, no previous reports describe the use of toehold biosensors for the direct detection of foodborne pathogenic bacteria in complex food matrices such as crude milk, particularly *L. monocytogenes*. In this study, we designed toehold biosensors targeting the V2 region of 16S rRNA from *L. monocytogenes* and selected the sensor that exhibited the highest stability and activation in response to a synthetic RNA trigger. We also demonstrate that this toehold can detect *L. monocytogenes* RNA derived from both pure cultures and artificially contaminated raw milk samples.

## 2 Materials and methods

### 2.1 Phylogeny and *in silico* trigger-target analysis

The V2–V3 fragment of the 16S rRNA gene from *L. monocytogenes* (GenBank accession number AJ535697.1), which corresponded to the target sequence selected for biosensor design, was used as the query to retrieve homologous sequences via BLASTn at the National Center for Biotechnology Information (NCBI, accessed 2025). All hits with significant similarity were included in the dataset to ensure representation of bacteria closely related to the biosensor target. To expand the taxonomic diversity of the analysis, an additional 16S rRNA sequence was manually curated, and its integrity was verified using reference strains from genera commonly reported in environmental, food-related, and clinical contexts, such as *Listeria*, *Bacillus*, *Streptococcus*, *Enterococcus*, *Lactobacillus*, *Paenibacillus*, *Escherichia*, and *Salmonella*. This expansion led to the selection of 110 sequences, which were aligned using Clustal Omega (Madeira et al., 2024). An alignment-curation process was then performed with Gblocks 0.91b through Phylogeny.fr interface (Dereeper et al., 2008), using default parameters to remove ambiguously aligned or gap-rich positions (Talavera and Castresana, 2007). Phylogenetic inference was conducted within a maximum-likelihood framework using PhyML 3.0 (Guindon and Gascuel, 2003), and branch support was evaluated with the SH-like approximation of the aLRT method. The resulting topology was visualized and annotated in iTOL v6 (Letunic and Bork, 2021) using a circular layout to facilitate examination of clade structure.

To evaluate whether the trigger region designed for the toehold biosensor (see the next section) displayed potential unintended complementarity with non-target bacterial sequences, we performed an alignment analysis. The 36-nt complementary trigger region, which corresponds to the trigger hybridization site that initiates stem opening in the toehold switch, was aligned against the V2-V3 16S rRNA sequences included in the phylogenetic dataset (*n* = 110). An initial BLASTn (NCBI) search was performed to detect full- or partial-sequence similarity. Subsequently, a multiple sequence alignment was generated with Clustal Omega, and the trigger- binding segment was retrieved from the alignment for comparative analysis. For each taxon, the number of matches, mismatches, and gaps within the trigger hybridization region was quantified to estimate the likelihood of off- target activation.

### 2.2 Toehold and trigger synthesis

The V2-V3 hypervariable region of the 16S rDNA from *L. monocytogenes* (Lm) was obtained from the NCBI BLASTn database^1^ and used to design the toehold switch sensor with the Nucleic Acid Package (NUPACK) (Zadeh et al., 2011), following the template for toehold design reported by Takahashi et al. (2018), using 37 °C and 1 mM NaCl as conditions (Zadeh et al., 2011). The sequences of the selected toehold switches and triggers, along with their NUPACK-predicted thermodynamic parameters, are provided in Supplementary Tables S1 and S2. In the template, the “source mRNA” was replaced with the V2-V3 region of *L. monocytogenes*, generating different toehold sensors. Three sensors, based on thermodynamic parameters, were selected and designated Toehold A, B, and C. The selected toehold sequences corresponded to the reverse complement of the trigger located in the V2 hypervariable region of the 16S rDNA from *L. monocytogenes*. Toehold switches were analyzed with SeqBuilder Pro (DNAstar Inc.) to confirm the absence of internal stop codons in the hairpin and to ensure they were in frame with the reporter gene. To enable T7 RNAP to drive the toehold and trigger *in vitro* transcription, a short nucleotide sequence from the plasmid vector and the T7 promoter were added at the 5′ end of the toehold sequence, and at the 3′ end, a sequence belonging to lacZ as a reporter gene. Likewise, sequences from the plasmid, T7 promoter, and T7 terminator were included at the 5′ and 3′ ends of the trigger, respectively. Toehold and trigger sequences with the additional elements were synthesized by Integrated DNA Technologies^2^ as double-stranded DNA fragments (gBlocks).

### 2.3 Toehold and trigger plasmid construction

To create the recombinant plasmids, we needed three different amplicons for assembly using Gibson (NEB E2611S). (i) A fragment containing the toehold or trigger sequences from *L. monocytogenes*, (ii) the lacZ gene, and (iii) the backbone of the pSNI_plasmid_sensor_LacZ (hereafter pSNI) (Addgene Inc., #139464) (Figure 1). The three fragments were amplified by PCR using overlapping primers to facilitate the assembly by a Gibson protocol (New England Biolabs, Catalog number E2611S). To obtain amplicons containing the toehold or trigger sequences, the DNA gBlocks were resuspended in nuclease-free water (final concentration of 10 ng/μL) and amplified by PCR with Phusion High Fidelity (NEB, M0531L) using the primers 3Fw-7Rv and 8Fw-7Rv, respectively. Amplification was performed in a C100 Touch Thermal Cycler (BioRad, Hercules, CA, United States), with the following conditions: 98 °C for 1 min, then 35 cycles of 30 s at 98 °C, 15 s at 60 or 64 °C, and 15 s at 72 °C, ending with a final extension at 72 °C for 5 min. The lacZ gene was amplified with primers 1Fw and 2Rv using this program: 98 °C for 1 min, followed by 35 cycles of 30 s at 98 °C, 30 s at 61 °C, and 2 min at 72 °C, then a final extension at 72 °C for 5 min. The backbone was amplified with primers 5Fw-6Rv (Supplementary Table S3) using 98 °C for 1 min, then 35 cycles of 30 s at 98 °C, 30 s at 69 °C, and 2.5 min at 72 °C, with a final extension at 72 °C for 5 min. Amplicons were visualized on 1% (p/v) or 2% (p/v) agarose gels and purified using a purification kit (NEB, T1030S). A Gibson master mix reaction (NEB, E2611S) containing the three amplicons was incubated at 50 °C for 30 min, placed on ice, and then transformed into NEB 5-alpha competent *E. coli* (NEB, C2987) Top10 cells supplemented with kanamycin (50 ng/mL). The cells were incubated at 37 °C overnight. Recombinant plasmids were extracted using a plasmid miniprep kit (NEB, T1010S), and the constructs were verified by PCR and sequencing. Recombinant plasmids harboring the toehold switch and trigger from *L. monocytogenes* sequence were named ToeholdA/pSNILacZ, ToeholdB/pSNILacZ, ToeholdC/pSNILacZ, TriggerA/pSNILacZ, TriggerB/pSNILacZ, TriggerC/pSNILacZ. As a control, a recombinant plasmid containing the toehold sensor and trigger sequences from *E. coli* (Takahashi et al., 2018) was constructed, following the strategy described above.

### 2.4 Toehold DNA template preparation and trigger RNA synthesis

Recombinant plasmids (Toehold B/pSNILacZ) and (Trigger/pSNILacZ) with a T7 promoter were amplified by PCR using Phusion High-Fidelity DNA polymerase (NEB, M0531L) or Q5 High-Fidelity DNA polymerase (NEB, M0491S) with primers 8Fw-4Rv (Supplementary Table S3). The resulting products were purified using the Monarch PCR & DNA cleanup kit (5 μg) (NEB T1030S) or the QIAquick PCR purification kit (Qiagen 28104). For trigger RNA synthesis, the purified amplicon was subjected to *in vitro* transcription using the HiScribe T7 High Yield RNA Synthesis Kit (NEB, E2040S). After 16 h (overnight) of incubation at 37 °C, the samples were treated with DNase I (RNase-free, NEB M0303S) for 15 min at 37 °C and then purified with the Monarch RNA Cleanup Kit (NEB, T2050). RNA triggers were quantified with a NanoDrop™ Lite spectrophotometer (Thermo Scientific) and used for toehold biosensor standardization and validation.

### 2.5 Validation of the toehold biosensors with synthetic triggers

Purified linear DNA (amplicon) containing the toehold-lacZ under the control of the T7 promoter, along with synthetic triggers, was used for toehold validation. Cell-free reactions were prepared on ice using the PURE Express *In Vitro* Protein Synthesis Kit (NEB, E6800S) in 11 μL reaction mixtures: 4 μL NEB Solution A, 3 μL NEB Solution B, 0.25 μL RNase inhibitor (Roche, 3335399001), 2.75 μL (1.6 nM) of linear DNA constructs encoding toehold switch sensors-lacZ, and 1 μL trigger RNA (5.286 μM), for a total volume of 11 μL. In this reaction, the final toehold:trigger ratio was approximately 1 nM:1000 nM. The mixture was incubated at 37 °C for 30 min, placed on ice for 5 min, and then 0.75 μL of 12 μg/μL CPRG (Sigma-Aldrich, 59767) was added. Samples were transferred to a 384-well plate (Corning, 3544) and incubated at 37 °C. LacZ expression was monitored at 576 nm using a SpectraMax i3 (Molecular Devices) at 2- or 4-min intervals for 1 to 4 h (Figure 1). Fold change was calculated by dividing the absorbance of the toehold activated with trigger by that of the toehold alone (Chakravarthy et al., 2021; Takahashi et al., 2018). When testing in 1.5 mL microcentrifuge tubes at a specific time, reactions were stopped with 2 M Na_2_CO_3_ before absorbance was measured at 576 nm.

### 2.6 Trigger RNA isolation from bacterial cultures

To evaluate the biosensor’s performance against natural bacterial targets, total RNA was isolated from 5 mL pure cultures of *L. monocytogenes* ATCC 15313 grown in TSB at 37 °C for 20 h. Two extraction methods were compared in the absence of a milk matrix: (i) a commercial method using the Monarch® Total RNA Miniprep Kit (NEB, T2010S), and (ii) an optimized TRIzol-based protocol described in the following section.

### 2.7 Toehold biosensor validation in raw milk

#### 2.7.1 Extraction of bacterial nucleic acids from raw milk

A 100 mL milk sample was collected from a healthy cow at the zootechnical station of the Life Science Division at the University of Guanajuato. The sample was manually collected into an RNase-free stool sample container and stored at 4 °C. The milk sample (25 mL) was then inoculated with 5 mL of an *L. monocytogenes* (ATCC 15313) inoculum (approximately 1 × 10^9^ CFU/mL) that had been cultured in tryptic soy broth (TSB) at 37 °C for 20 h. The purity of the milk matrix was verified using a molecular quality-control protocol to exclude endogenous *L. monocytogenes*. Nucleic acids (DNA) were extracted from non-inoculated milk samples (matrix control M) and from inoculated milk containing *L. monocytogenes* according to the Volk protocol (Volk et al., 2014) and subjected to PCR assays using specific primers to detect *L. monocytogenes* (Supplementary Table S3). The absence of amplification in non-inoculated milk samples, compared with the clear bands observed in inoculated positive controls, confirmed that the raw milk was free of the target pathogen and that the recovery method is highly selective, preventing cross-reactivity with native milk DNA.

#### 2.7.2 Bacterial RNA extraction from raw milk

Once it was confirmed that the milk was free of *L. monocytogenes*, bacterial RNA was isolated using an optimized TRIzol-based protocol (Invitrogen, 15596026) to handle the high lipid and protein content of dairy samples (Carballo-Uicab et al., 2026). To evaluate the method’s scalability and efficiency, variable volumes of *L. monocytogenes* (ATCC 15313) inoculum in tryptic soy broth (TSB), ranging from 2 to 10 mL (each pellet containing ~2 × 10^9^ to 1 × 10^10^ CFU), were grown at 37 °C for 20 h. The resulting pellets were resuspended in a 1 mL milk aliquot, achieving a final density of ~4 × 10^7^ CFU/mL (equivalent to an OD600 of 0.04) for biosensor validation. Samples were centrifuged at 18000g for 10 min (Microcentrifuge Science MED D1524R), and the bacterial pellets were resuspended in 1 mL of fresh raw or pasteurized milk, reaching approximately 4 × 10^7^ CFU/mL (OD600 of ~0.04). Next, the pellets were centrifuged again at 16000g for 20 min. After centrifugation of the inoculated milk, the upper fat layer was manually removed with a sterile cotton swab to prevent lipid interference. The resulting pellets underwent sequential washes with PBS supplemented with 0.5 mM EDTA to effectively displace caseins and residual proteins. Following enzymatic lysis with lysozyme (0.32 mg/mL), standard TRIzol™ phase separation was performed, followed by triple washes of the RNA pellet with 75% (v/v) ethanol to remove residual contaminants. These modifications significantly improved the A260/280 ratios, ensuring RNA of sufficient purity for biosensor validation. Total RNA quality and quantity were assessed using a Nanodrop Lite spectrophotometer (Thermo Scientific). RNA quality was estimated by calculating the A260/280 ratios, and concentration was determined by measuring absorbance at 260 nm. Finally, the products were visualized by gel electrophoresis on a 1.2% agarose gel. All RNA samples were treated with the DNase Monarch Spin RNA Isolation Kit (Mini) (NEB T2110) and purified with the Monarch Spin RNA cleanup Kit (T2050S) according to the manufacturer’s instructions. As a negative control, raw milk was deliberately inoculated with *E. coli* Top 10 using a matching biomass load (a pellet from 2 mL of stationary-phase culture resuspended in 1 mL of milk). This ensured that the complexity of the total RNA extracted was comparable to the target samples, allowing for a rigorous evaluation of the biosensor’s specificity against non-target bacterial RNA.

#### 2.7.3 Detecting *L. monocytogenes* in raw milk with toehold biosensors

A linear DNA construct encoding the toehold switch B-lacZ was used as the sensing template to detect RNA triggers extracted from the following samples: raw milk inoculated with *L. monocytogenes* (LmM), raw milk inoculated with *E. coli* (EcM), and raw milk without bacterial inoculum (M) as a matrix control. Other controls included RNA extracted from pure cultures of *L. monocytogenes* (LmP), *E. coli* (EcP), and water used in the experiment. Cell-free reactions were assembled on ice using PURE Express (NEB, E6800S). To determine the optimal activation conditions, we tested different trigger concentrations, and the best results were obtained as follows: NEB Solution A (2 μL) (40%), NEB Solution B (1.5 μL) (30%), 0.12 μL of RNase inhibitor (Roche, 3335402001), 0.98 μL (3 nM) of linear DNA constructs encoding toehold switch sensors-LacZ, and 0.45 μL (1.8 μM) of trigger RNA, with a total reaction volume of 5.05 μL. In this reaction, the final concentration of toehold:trigger was approximately 1 nM:276 nM. Samples were incubated for 2 hours at 37 °C, then placed on ice for 5 min. Subsequently, 0.3 μL of 12 mg/mL CPRG (Sigma-Aldrich, 59767) was added, and the mixture was incubated at 37 °C in the Synergy HTX model (HTXS1LFA) with integrated BioTek Gen5 software (version 2.06) to read the absorbance of a black 384-well plate (Corning, 3544) at 576 nm every 4 min for 2 h (Supplementary Figure S1).

### 2.8 Statistical analysis

Data processing, statistical analyses, and visualizations were conducted using Python (version 3.12). The pandas library (version 3.0.3) was used for dataset manipulation and calculation of endpoint fold-changes. To evaluate the statistical significance of differences among experimental groups, a one-way analysis of variance (ANOVA) was performed using the SciPy library (version 1.18.0), followed by Tukey’s Honestly Significant Difference (HSD) *post hoc* test for multiple pairwise comparisons using the statsmodels library (version 0.14.6). A *p*-value <0.05 was considered statistically significant. Graphical representations, including a compact letter display to denote significant differences and standard deviation error bars, were generated using the Matplotlib (version 3.11.0) and seaborn libraries (version 0.13.2).

## 3 Results

### 3.1 Phylogeny and *in silico* trigger-target analysis

**Figure 2:**
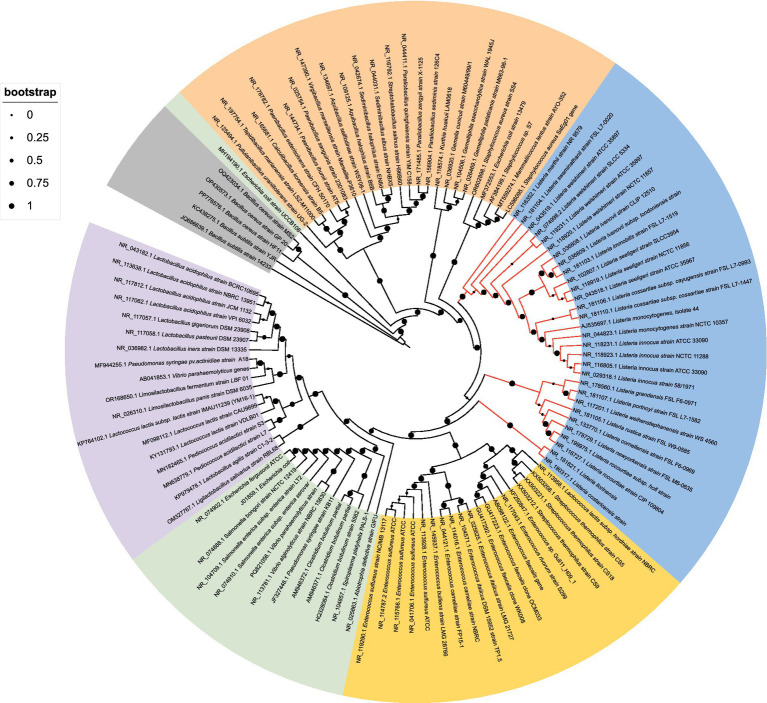
Phylogenetic analysis of the V2-V3 hypervariable regions of 16S rRNA from *Listeria species* and related bacteria. A phylogenetic tree was constructed from an alignment of the V2-V3 region of the 16S rRNA gene, including *Listeria monocytogenes* and bacterial taxa commonly associated with dairy products. Lactic acid bacteria (purple), Gram-negative dairy-associated bacteria (green), food-related reference taxa (yellow), and *Bacillus species* (orange) were included to evaluate potential cross-reactivity. *Listeria* strains form a well-supported monophyletic clade (blue). *Bacillus subtilis* (JQ686639.1) served as the outgroup to root the tree. Accession numbers for all sequences are provided.

A maximum-likelihood phylogeny based on 110 sequences of the V2-V3 region of the 16S rRNA gene revealed a well-supported monophyletic clade that includes all *Listeria* spp. in the analysis. The Listeria spp. clade displayed short internal branch lengths and high SH-like aLRT support values (0.75–1.0), indicating low sequence divergence within the genus (Figure 2). Non-*Listeria* sequences were resolved as distinct clades corresponding to *Bacillaceae*, *Lactobacillales*, *Actinobacteria*, *Enterobacterales*, and *Pseudomonadales*. These groups exhibited longer branch lengths than Listeria spp., reflecting substantial divergence in the V2–V3 region across bacterial groups. No non-Listeria sequences clustered near the Listeria clade, confirming the presence of phylogenetically informative signatures unique to this genus. This phylogenetic structure supports the use of the V2-V3 region as a molecular target for discriminating Listeria spp. and validates its suitability for developing a sequence-specific RNA biosensor.

### 3.2 In silico trigger specificity analysis

**Figure 3:**
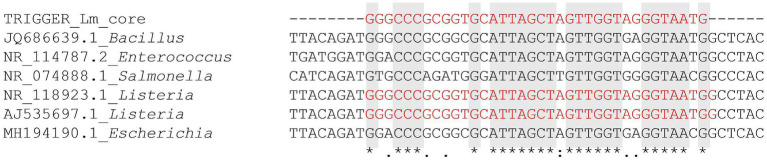
Multiple sequence alignment of the 36-nt trigger of toehold B against representative V2-V3 16S rRNA sequences. The trigger region is fully conserved in *Listeria monocytogenes* (NR_118923.1 and AJ535697.1), while other taxa (*Bacillus*, *Enterococcus, Salmonella*, and *Escherichia*) show multiple mismatches within the target window. Asterisks indicate conserved positions. These data support the biosensor’s high sequence specificity.

To evaluate the biosensor’s sequence-level specificity, a 36-nt core fragment of the trigger, derived from the V2-V3 region of the *L. monocytogenes* 16S rRNA gene, was analyzed. A BLASTn alignment against the 16S sequence of *L. monocytogenes* isolate 44 (AJ535697.1) revealed a perfect identity (36/36 nt, 100% identity), confirming that the synthetic trigger accurately reproduces the native target sequence. A total of 110 V2-V3 sequences from milk-associated and environmental bacteria were then scanned with this 36-nt trigger core. Only *Listeria* spp. showed a perfect match, whereas non-*Listeria* taxa displayed 1 to 19 mismatches within the trigger. A focal alignment with representative taxa (*Listeria*, *Bacillus*, *Enterococcus*, *Salmonella*, and *Escherichia coli*) showed complete conservation of the trigger region in *Listeria* spp., whereas non-target bacteria exhibited multiple mismatches, particularly at positions critical for toehold hybridization. These results suggest that off-target activation is unlikely at the sequence level, although some background hybridization cannot be completely ruled out due to the inherent leakiness of toehold systems (Figure 3).

### 3.3 Design, construction, and initial specificity testing of candidate toehold sensors

**Figure 4:**
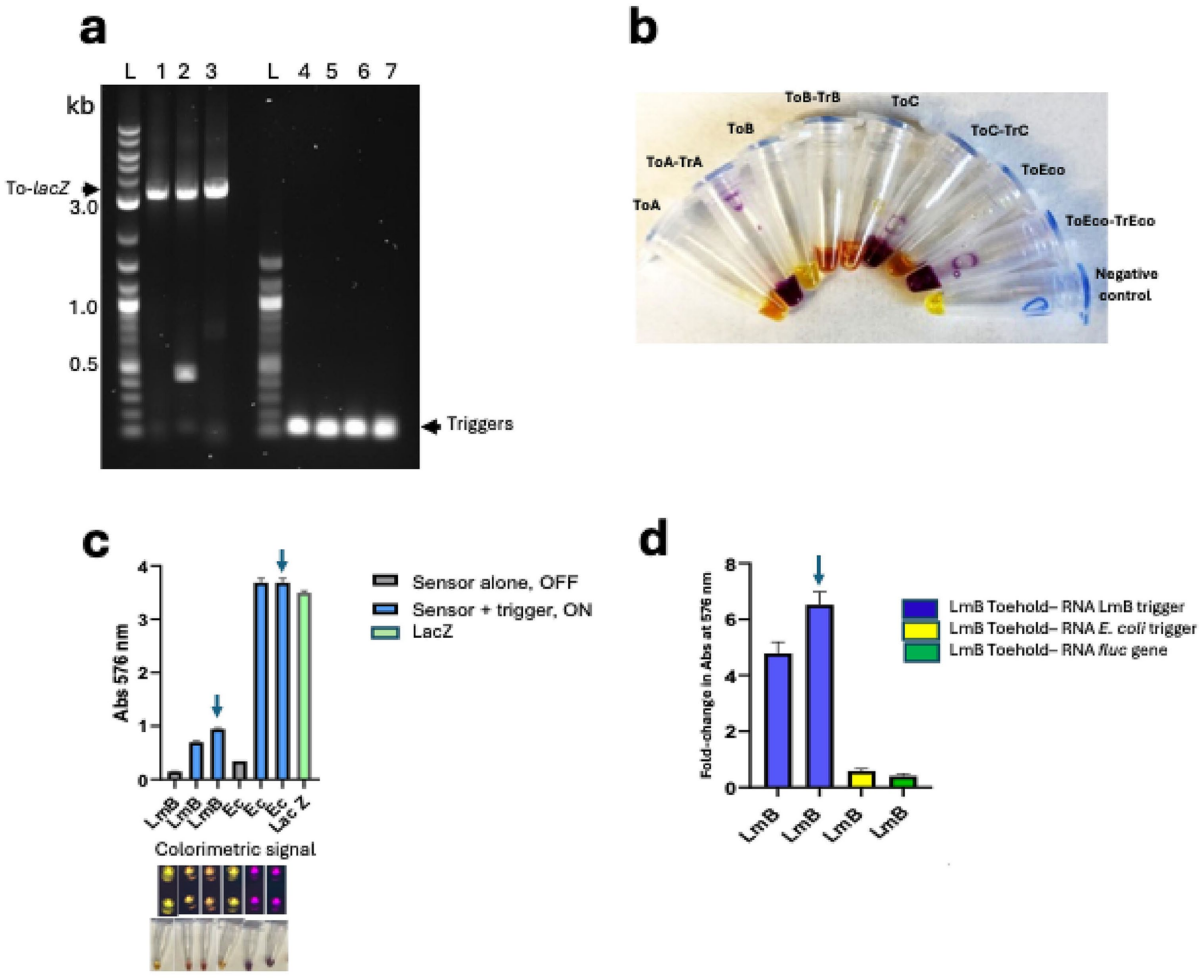
Validation of the toehold/pSNI and trigger/pSNI construction, activation, and specificity. **(a)** The toehold-lacZ (To-lacZ, shown with an arrow on the left) was amplified from toeholdA/pSNI, toeholdB/pSNI, and toeholdC/pSNI, producing amplicons of approximately 3,300 bp (lines 1, 2, 3). Triggers from triggerA/pSNI, triggerB/pSNI, triggerC/pSNI, and trigger/pSNI from *E. coli* (Ec) were amplified as PCR fragments of about 190 bp (lines 4, 5, 6, 7) (shown with an arrow on the right). **(b)** Different toeholds (Toehold A, ToA; toehold B, ToB; toehold C, ToC) from *L. monocytogenes* and toehold from *E. coli* (ToEc) were activated with their corresponding triggers, generating LacZ that hydrolyzes CPRG (yellow), resulting in various colors (orange, red, purple) depending on LacZ concentration. **(c)** Toehold B (LmB) from *L. monocytogenes*, and toehold derived from *E. coli* (Ec) were activated by RNA triggers synthesized using recombinant plasmids or the gBlock (blue vertical arrow) as templates. As a control, LacZ was amplified from pSNI. Absorbance was measured after 5 min of incubation at 37 °C following the addition of CPRG in a cell-free reaction. **(d)** Specificity of toehold B (LmB). The toehold is activated (ON state) in the presence of its specific RNA trigger, produced with recombinant plasmids or the gBlock (blue vertical arrow) as templates. When toehold B was exposed to triggers from *E. coli* or the *fluc* gene, the sensor remained deactivated (OFF state). Absorbance was measured 30 min after incubation at 37 °C following the addition of CPRG to a cell-free reaction. In **(c,d)**, data are presented as the mean ± standard deviation. The mean and standard deviation were calculated from the deviations and represent the average of four independent assays, each with triplicate measurements. Fold change was calculated by dividing the absorbance of the toehold activated with the trigger by the absorbance of the toehold.

Ten toehold switch sequences and triggers were designed *in silico* using NUPACK. Based on free-energy folding and the presence of internal stop codons that could block translation of the reporter protein, three candidates were selected and named toehold A (Δ*G* = −34.95 kcal/mol), toehold B (Δ*G* = − 42.28 kcal/mol), and toehold C (Δ*G* = −38.35 kcal/mol), along with their respective triggers (Supplementary Tables S1, S2). These toeholds and triggers were assembled downstream of lacZ in the pSNI plasmid and placed under the control of the T7 promoter and T7 transcriptional terminator. The presence of toeholds and triggers in the recombinant plasmids was confirmed by PCR, yielding amplicons of approximately 190 bp and 3,290 bp, respectively. The next step was to evaluate whether the candidate toehold sensors are activated in the presence of the triggers (ON state) and deactivated in their absence (OFF state). We observed that toehold B was activated by its specific trigger (i.e., trigger B), regardless of whether it was synthesized from DNA obtained directly from gBlocks or from the recombinant plasmid trigger/pSNI. When toehold B was exposed to triggers from *E. coli* and the firefly luciferase (*fluc*) gene, it remained in the OFF state (Figure 4). Similar results were obtained for toeholds A and C, indicating that the three candidate sensors showed trigger-dependent activation under the tested conditions.

### 3.4 Functional comparison and selection of the toehold with the highest activity and stability using a synthetic trigger

**Figure 5:**
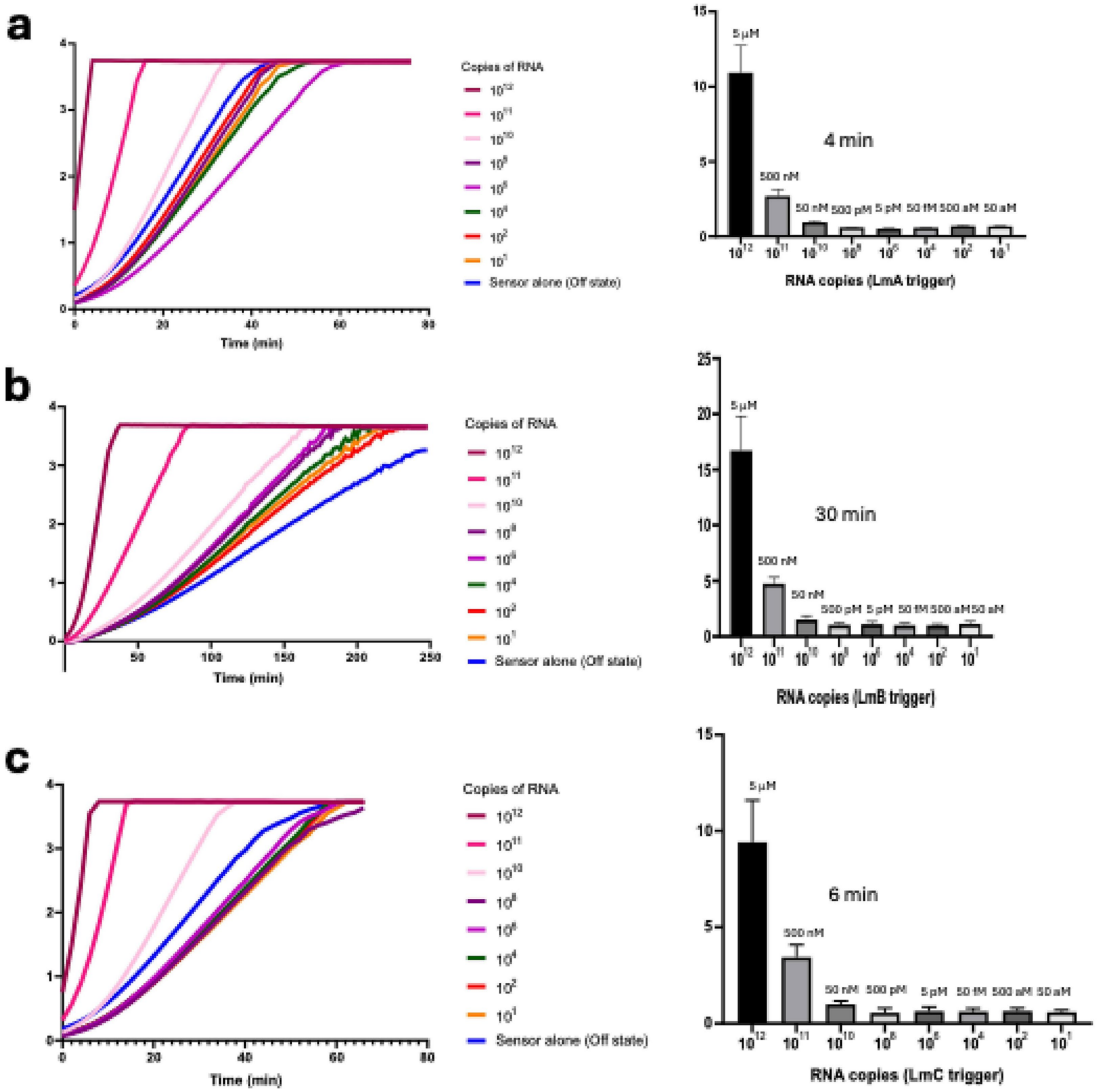
Toehold activation with multiple copies of synthetic RNA triggers at different times. **(a)** Toehold A, **(b)** Toehold B, and **(c)** Toehold C were activated with synthetic RNA triggers A, B, and C, respectively. On the left, 0.4 nM toehold (final concentration) was activated with different copies of their corresponding triggers. On the right, the fold-change was calculated at the time of the highest value of the fold change (see Figure 6). Data are presented as the mean ± standard deviation. The mean and standard deviation were calculated from the deviations and are presented as the average of four independent assays, each with triplicate measurements.

**Figure 6:**
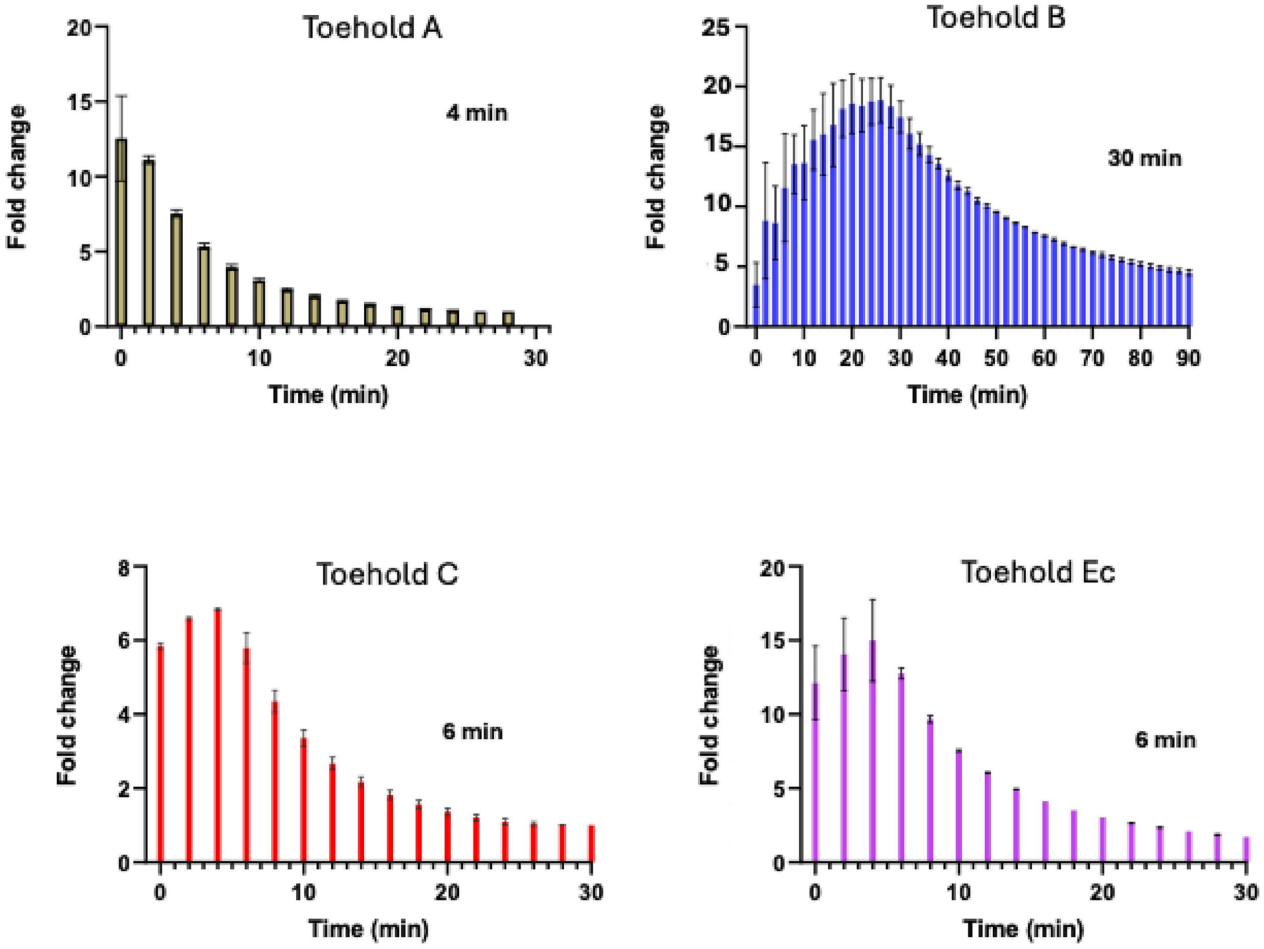
Determination of toehold fold changes over time involved measuring absorbance at 576 nm. A final concentration of 0.4 nM toehold was activated with 10^12^ synthetic RNA triggers. CPRG was used as a substrate for LacZ, as described in the materials and methods. Data are presented as the mean ± standard deviation. The mean and standard deviation were calculated from the deviations and are presented as the average of four independent assays, each with triplicate measurements.

After demonstrating that toeholds are specific to their corresponding targets, the next step was to determine which sensor shows the highest activation and stability, compare candidate sensors, and evaluate concentration-dependent signal behavior. The three Toehold sensors were tested with synthetic RNA trigger inputs ranging from 10^1^ to 10^12^, corresponding to approximately 50 aM to 5 μM. The three toeholds were tested with RNA trigger inputs down to 50 aM; however, at this concentration, the activation ratios were close to 1, indicating minimal activation above background. However, their maximum fold-changes for toeholds A and C were approximately 10, achieved at 4 and 6 min with 10^12^ copies of the RNA trigger (5 μM). Toehold B reached activation ratios of approximately 17 and 5 with 10^12^ and 10^11^ RNA copies, i.e., 5 μM and 500 nM, respectively. Toeholds A and C (without the triggers) were less stable than toehold B, as they reached their maximum absorbance approximately at 40 min, indicating that their stem-loop structures are completely open in the absence of the trigger (Figures 5, 6). Based on these data, the three toeholds could be considered good biosensors, as a 5-fold minimum activation ratio is required to achieve the desired sensitivity (Chakravarthy et al., 2021; Takahashi et al., 2018); nevertheless, we decided to select toehold B for further studies because of its highest stability and activation ratio (fold-change).

### 3.5 Sensor activation with bacterial RNA

Before evaluating the toehold B with bacterial RNA, we tested toehold activation using various concentrations of synthetic RNA trigger. The goal was to standardize the assay and identify a working RNA concentration that consistently activated toeholds in subsequent assays, using bacterial RNA from either pure cultures or from milk samples contaminated with bacteria.

**Figure 7:**
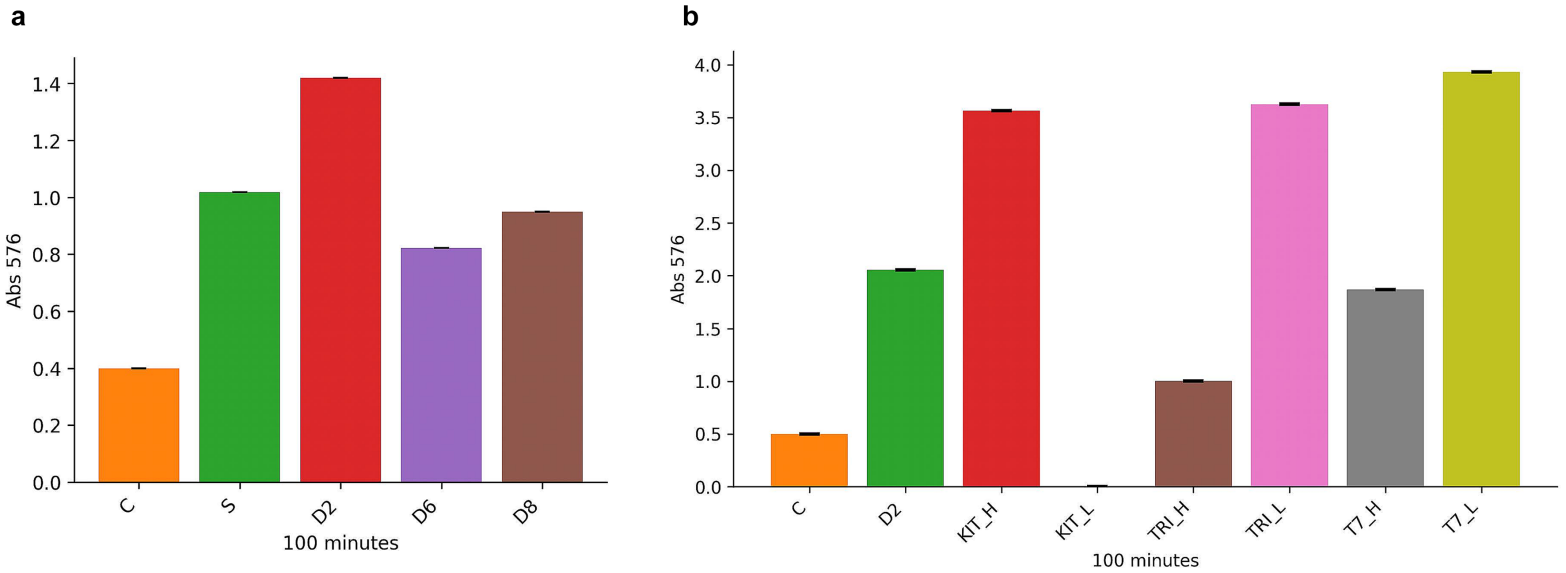
Optimal condition of the activation of toehold B with different RNA triggers. **(a)** Absorbance curves (OD576) of the toehold B in PURExpress® reactions done in 5.05 μL, showing the response to serial dilutions of the synthetic RNA trigger. The plot includes the negative control (C, no RNA/no trigger), the biosensor without added RNA (ToB, 3 nM), the stock solution (S, 7.158 μM), and specific dilutions: D2 (1.78 μM), D6 (0.1118 μM), and D8 (0.0279 μM). **(b)** Comparative evaluation of the biosensor response to different RNA sources: synthetic RNA controls (T7_H (72.2 μM), T7_L (1.44 μM)) produced via *in vitro* transcription; total RNA extracted from *L. monocytogenes* pure cultures using a commercial kit (KIT_H (1.18 μM), KIT_L (0.059 μM) or TRIzol (TRI_H (7.39 μM), TRI_L (1.84 μM); and the synthetic positive control (D2, 1.78 μM). Negative controls included biosensor only (ToB, 3 nM), and the reaction mix without biosensor or trigger (C). The photographs above the plot display the final visual endpoint of the reactions, aligned with the order shown in the graph. Data are presented as the mean ± standard deviation. The mean and standard deviation were calculated from the deviations and are presented as the average of three independent assays, each with duplicate measurements.

A dilution series of the synthetic trigger, including the stock solution and dilutions (d1–d8), ranging from 7.158 μM to 0.0279 μM, was tested. As shown in Figure 7a, the ToB biosensor, used at a concentration of 3 nM, displayed a clear concentration-dependent response. The most robust and consistent activation was observed at a trigger concentration of approximately 1.78 μM (D2), with an absorbance of 1.4. Higher trigger (RNA) concentrations (stock S, 7.158 μM) had absorbance values similar to those of D6 (0.1118 μM) and D8 (0.0279 μM), with absorbances around 1 (Figure 7a). Subsequently, we evaluated the biosensor’s response to biological RNA preparations. This step is essential for ensuring reproducibility because biological RNA has secondary structure and may contain cellular contaminants that are absent from synthetic oligonucleotides. Following the initial calibration of the biosensor with synthetic RNA (Figure 7a), we assessed its performance using trigger (RNA) derived from three methods: (i) *in vitro*-transcribed RNA using HiScribe™ T7 High Yield RNA Synthesis at two concentrations levels, T7_H (72.2 μM) and T7_L (1.44 μM), (ii) total RNA purified with a commercial Monarch® Total RNA Miniprep Kit, KIT_H (1.18 μM) and KIT_L (0.059 μM), and (iii) RNA extracted using TRIzol®, TRI_H (7.39 μM) and TRI_L (1.84 μM). All samples were compared with the previously established D2 concentration (1.78 μM) (Figure 7b). The three RNA preparation methods were functional in PURE Express system. This indicates that the biosensor response is influenced by the RNA preparation method, likely due to differences in RNA purity, secondary structure, or residual components, yet remains detectable when RNA inputs are within the selected working range.

Additionally, the analysis revealed that higher trigger RNA concentrations were associated with lower activity, and lower concentrations of TRI_L (1.84 μM), KIT_L (0.059 μM), and T7_L (1.44 μM) produced the highest activity with absorbance of approximately 3.5 (Figure 7b). However, KIT_L (0.059 μM) maintained sustained activity to saturation, exceeding the Synergy software’s detection limits. The negative control (C, no ToB/RNA added) remained flat, confirming the absence of spontaneous activation. The internal design control ToB (3 nM) (no RNA added) showed its characteristic basal kinetics, serving as a baseline for comparison. These results show that the biosensor remains reliably activated by *Listeria* RNA extracted with either a column-based kit or TRIzol®, reaching maximum absorbance of approximately 3.5 with triggers of 1.18 μM and 1.84 μM, respectively (Figure 7b).

### 3.6 Toehold biosensor detection of *L. monocytogenes* in raw milk

**Figure 8:**
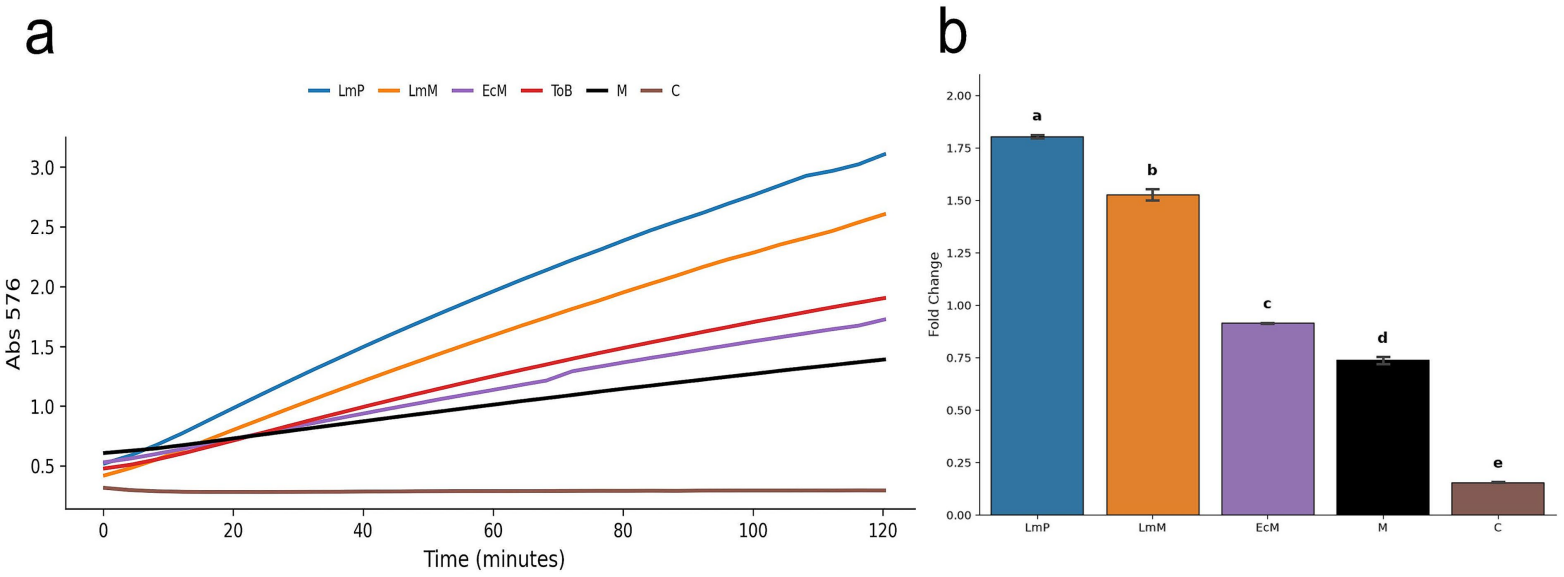
Detection of *Listeria monocytogenes* in raw milk using toehold B biosensors. **(a)** Time-course absorbance of the toehold biosensor in PURExpress reactions with RNA extracted by TRIzol from a pure culture of *L. monocytogenes* (LmP), *L. monocytogenes* inoculated into raw milk (LmM), *E. coli* inoculated into raw milk (EcM), raw milk without inoculum (M), all samples in the same concentration (1.84 μM), the biosensor without RNA (3 nM ToB), and a mix reaction without toehold and trigger **(b)**. Data are presented as the mean ± standard deviation from three independent assays, each performed in duplicate. Fold-change values were calculated relative to the Toehold B control at 120 min. Statistical significance was assessed using one-way ANOVA with Tukey’s multiple-comparison test. Different letters indicate statistically significant differences (*p* < 0.05). The mean and standard deviation were calculated from the deviations and are presented as the average of three independent assays, each with duplicate measurements.

Bacterial RNA extraction from inoculated milk was optimized using TRIzol. Approximately 1.84 μM was established as the optimal bacterial trigger RNA concentration for TRI-L, compared with 1.78 μM for the synthetic trigger (Figure 7). The biosensor was tested with RNA extracted from raw milk inoculated with *L. monocytogenes* (LmM), *E. coli* (EcM), and control raw milk without a bacterial inoculum (M). The control included RNA from pure cultures of *L. monocytogenes* (LmP). Because an *E. coli* toehold was used as a control during the initial testing of sensors with synthetic trigger RNA, we maintained continuity by using *E. coli* as a control as well, rather than employing common foodborne pathogens found in raw milk. Toehold B without RNA (3 nM ToB) served as the biosensor-only background control, whereas a reaction mixture without toehold and trigger (C) served as the PUREexpress reaction background control. The time-course assay showed that RNA from a pure *L. monocytogenes* culture (LmP) produced the highest absorbance, followed by RNA extracted from *L. monocytogenes*-contaminated raw milk (LmM) (Figure 8a). The matrix-associated samples, including RNA from *E. coli*-contaminated milk (EcM) and non-inoculated raw milk (M), showed lower absorbance than LmP and LmM. At 120 min, the absorbance values of EcM and M were lower than those of the Toehold B-only control lacking RNA (EcM: Abs 1.72; M: Abs 1.39; ToB: Abs 1.90), suggesting the absence of strong target-dependent activation in these samples under the tested conditions. The reaction mixture without toehold and trigger (C) showed the lowest background (Figure 8a).

Endpoint fold-change values at 120 min were calculated relative to Toehold B-only and are shown in Figure 8b. RNA from pure bacterial cultures of *L. monocytogenes* (LmP) yielded an endpoint fold-change of approximately 1.8, whereas *L. monocytogenes*-derived RNA extracted from contaminated raw milk (LmM) yielded an endpoint fold-change of approximately 1.5. For RNA extracted from *E. coli*-contaminated milk (EcM), the endpoint fold-change was approximately 0.9, whereas RNA from non-inoculated raw milk (M) produced an endpoint fold-change of approximately 0.73. Endpoint fold-change values were analyzed using one-way ANOVA with Tukey’s multiple-comparison test. The analysis showed significant differences in activation rates among samples from bacterial cultures of *L. monocytogenes* (LmP), raw milk contaminated with *Listeria* (LmM), crude milk (M), and raw milk contaminated with *E. coli* (EcM) (Figure 8b, Supplementary Table S4).

## 4 Discussion

To expand options for detecting *L. monocytogenes*, we aimed to design, build, and test toehold biosensors capable of identifying this bacterium not only with synthetic triggers but also with total bacterial RNA from pure cultures or from food matrices (i.e., raw milk) contaminated with the bacterium. Results showed that toehold biosensors have a high activation rate with synthetic triggers and exhibit low activation with total RNA from pure cultures or contaminated raw milk without prior enrichment of the trigger region; however, activation is sufficient to distinguish *Listeria* from signals generated by RNAs in milk.

At the outset, a non-virulent gene region was selected as a specific trigger for *L. monocytogenes* detection. We chose the 16S rRNA gene because sequences within the V2-V3 hypervariable region of this housekeeping gene have been shown to be effective for detecting the human gut microbiota, distinguishing *Listeria* spp. from other bacteria, and providing the highest resolution for differentiating lower taxonomic levels, such as genera and species (Barboza-Corona et al., 2026; Bukin et al., 2019; Takahashi et al., 2018). *In silico* analysis of the V2-V3 region grouped *L. monocytogenes* into a monophyletic clade (Figure 2), suggesting that this region may be useful for detecting any *Listeria* species, as previously reported for the V2 region (Barboza-Corona et al., 2026), supporting the suitability of the chosen region as a specificity marker. This region (V2-V3) was used to design toehold switches and triggers, but energetic predictions with NUPACK showed that the most stable were three toeholds (called A, B, C), whose triggers belong to the V2 region. Notably, a 36-nt trigger sequence that activates toehold B showed perfect identity only with *Listeria* spp., whereas non-*Listeria* bacteria exhibited multiple mismatches or short homology patches (Figure 3). This is important because a single mutation in the trigger sequence can reduce switch activation, as demonstrated elsewhere (McSweeney et al., 2023; Ekdahl et al., 2022).

It was observed that the toehold B was orthogonal to its corresponding triggers and remained OFF when exposed to the *E. coli* trigger (Figure 4). The orthogonality of the toehold biosensors is critical for specificity, meaning only the correct RNA trigger sequence unlocks the hairpin, exposes the ribosome-binding site, and allows the translation of a reporter protein (McSweeney et al., 2023; Takahashi et al., 2018; Pardee et al., 2016; Green et al., 2014). The same orthogonality behavior was observed for toeholds A and C. Toeholds A and C also respond more quickly than toehold B, indicating rapid unfolding upon contact with the synthetic trigger. Although toehold B activates more slowly, it is more thermodynamically stable: toeholds A and C showed leakage, with the hairpin structure opening spontaneously around 40 min, whereas toehold B takes up to 200 min to fully relax and reach maximum activation without the trigger (Figure 5). Several studies have reported varying activation times for toeholds in cell-free systems, which depend on the sequence, stability, and toehold concentrations, as well as on the concentration and nature of the trigger used in the assay. For example, Diaz et al. (2025) reported toehold activation within 17–25 min, while Chakravarthy et al. (2021) and Pardee et al. (2014) observed activation times ranging from minutes to an hour. Activation of the three biosensors in this work falls within this timeframe, with toehold B’s 30-min response matching most closely. At peak activation, all three toehold switches showed a 10- to 15-fold change, which is within the 2-15-fold range reported for detecting 10^12^ RNA copies of SARS-CoV-2 without NASBA amplification (Chakravarthy et al., 2021). Other studies have reported fold changes of approximately 80 using 3,000 nM of synthetic triggers, such as in the activation of Ebola toehold sensors (Pardee et al., 2014). Additionally, biosensor activation depends on the concentrations of the toehold and trigger. For example, at 3 nM toehold used in this work, the fold change is higher with a 1.78 μM synthetic trigger (2.3-fold) than with 7.158 μM or 0.0279 μM (1.5-fold and 1.3-fold, respectively) (Figure 7). This suggests that activation of the biosensor (toehold B) is highly dependent on the concentration of the trigger (RNA) input and that the response is not strictly proportional to the amount of trigger added. At low trigger concentrations, the number of productive toehold switch–trigger interactions may be insufficient to efficiently open the toehold structure and expose the ribosome-binding site. In contrast, excessive RNA input may reduce reporter output by promoting nonproductive RNA interactions, altering trigger accessibility through secondary structure, increasing molecular crowding, or imposing an additional burden on the cell-free transcription–translation reaction. This concentration-dependent behavior explains why high analyte concentrations did not necessarily produce stronger activation and why RNA input optimization was required before testing bacterial RNA and milk-derived RNA samples. Similar concentration-dependent effects have been reported in previous toehold-based systems (Morey et al., 2023; Arce et al., 2021; Green et al., 2014; Pardee et al., 2016).

The next step was to test whether the toehold switch B could be activated by RNA extracted from a pure culture of *L. monocytogenes*. Using *Listeria* RNA implies that the sequence (trigger) that activates the toehold, present in the 16S rRNA, will compete with other RNAs (mRNA, tRNA, and other rRNAs) of different sizes (e.g., 75–90 nt for tRNA and about 1,500 nt for 16S rRNA) (Větrovský and Baldrian, 2013) or with secondary structures that could hinder interaction with the toehold (Pardee et al., 2014; Větrovský and Baldrian, 2013). Typically, synthetic triggers that activate toeholds are 36 nt in size, but triggers of 18–22 nt have also been reported as useful for activation (McSweeney et al., 2023; Pardee et al., 2014). To our knowledge, only one study has used a large synthetic trigger of 5,100 nt to activate toeholds for SARS-CoV-2 detection, achieving approximately a 1.75-fold change at trigger concentrations ranging from 1 fM to 100 nM (Morey et al., 2023). Although it is a large trigger, it is probably less complex than a mixture of RNAs from a bacterium. We tested various protocols for extracting bacterial RNA, including the RNA extraction method for buffalo milk (Sharma et al., 2018), Volk et al. (2014), and the Monarch Total RNA Miniprep Kit. However, some of these methods require larger sample sizes, are time-consuming, and are not compatible with cell-free reactions. Although the commercial kit is useful for extracting bacterial RNA from pure cultures, it was ineffective for extracting RNA from raw milk. We then developed a TRIzol-based protocol, a column-free kit workflow specifically designed for RNA extraction from both sample types (Carballo-Uicab et al., 2026). This method eliminated the need for large amounts of material and for a column kit; importantly, the RNA samples remained compatible with the cell-free system assay (Figure 7). When we used 1.84 μM of *Listeria* RNA (trigger) obtained by TRIzol (TRI_L), it showed the same absorbance as 1.18 μM of *Listeria* RNA (trigger) obtained with a commercial kit (KIT_H), around 3.5 (Figure 7b), indicating that bacterial RNA extraction with TRIzol is effective for cell-free reactions. Interestingly, using 1.84 μM RNA obtained with TRIzol (TRI_L) as a trigger resulted in a 2.6-fold change, which is higher than the 1.75-fold change reported with a large synthetic trigger of 5,100 nt from SARS-CoV-2 (Morey et al., 2023). The activation of toehold B with *Listeria* RNA is notable, as the trigger region in the 16S rRNA competes with other RNAs in the sample, yet it still activates the toehold. To our knowledge, this is the first time a complex RNA sample has been used as a trigger to activate a toehold switch sensor.

In the subsequent step, the toehold-trigger assay was performed using total RNA obtained from milk samples. Milk is a complex matrix food that, in addition to its chemical composition, may contain endogenous bacteria and somatic cells (Luo et al., 2024; Xie et al., 2024; Yuan et al., 2022; Powell et al., 1994). This indicates that milk RNA contains RNA from native cells and bacterial RNA from *L. monocytogenes* when the milk is artificially contaminated, making the sample more complex than pure bacterial RNA or synthetic triggers. When the assays were performed, the sensor showed a higher activation ratio with RNA from a pure culture of *L. monocytogenes* (LmP), with a fold change of approximately 1.8 In contrast, RNA from milk contaminated with *Listeria* (LmM) had a fold change of about 1.5, which showed a significant difference (Figure 8b, Supplementary Table S4). The lower fold-change with RNA from *L. monocytogenes*-contaminated milk (LmM) compared to pure culture (LmP) likely reflects the greater complexity of the raw-milk-derived RNA sample. In pure culture, RNA is enriched for bacterial RNA from the target organism, while in contaminated milk, *Listeria* RNA is mixed with matrix components, endogenous microbial or somatic-cell RNA, non-target RNA, and residual extraction compounds. Residual lipids, proteins, salts, calcium, organic compounds, or milk-derived inhibitors may reduce enzymatic efficiency, increase variability, or alter cofactors (Acquavia et al., 2025). These factors may decrease the target sequence’s abundance or accessibility and partially impact downstream transcription–translation efficiency. RNA extracted from *L. monocytogenes*-contaminated milk (LmM) showed activation, indicating that the TRIzol method was compatible with cell-free reactions. However, not all inhibitory components from the matrix or the extraction were removed, which may still cause background variability or reduced efficiency, as seen in reactions with complex samples (Schrader et al., 2012; Sidstedt et al., 2020). Thus, optimizing RNA cleanup, input, endpoint selection, and toehold design is necessary to improve signal-to-background and discrimination from controls.

When comparing the activation ratio of toehold sensing *Listeria* present milk (1.5-fold change) with that of studies using NASBA-amplified synthetic triggers to activate specific toeholds for the Ebola virus (fold-changes >20) (Pardee et al., 2014), the activation ratio is lower. However, this is in the range of the activation ratio for detecting SARS-CoV-2 without NASBA using a trigger RNA obtained from a nasopharyngeal sample (i.e., 1), but lower when the trigger is enriched by NASBA (i.e., 4) (Chakravarthy et al., 2021). The concentration of 1.84 μM RNA trigger used to activate the toehold in the milk assay is low compared with trigger concentrations used in other studies to detect different systems, such as the Zika virus (Pardee et al., 2016, 2014; Addgene Blog, 2016; Green et al., 2014). Although sensitivity remains limited, toehold switch sensors are functional in samples derived from food matrices. One of the most important findings in this work is that the toehold B can be activated by a trigger that competes with a highly complex system composed of different types of RNAs. NASBA is a protocol that has proven useful for enriching the trigger sequence and boosting sensitivity to attomolar or femtomolar RNA concentrations (Chakravarthy et al., 2021; Takahashi et al., 2018; Pardee et al., 2014). One of our first future challenges will be to determine whether NASBA (Takahashi et al., 2018) or another method, such as TEV (Morey et al., 2023), can effectively increase sensitivity for detecting *L. monocytogenes* in raw milk by enriching the trigger region, given that during TRIzol RNA extraction, the sample may also contain mRNA, tRNA, and rRNA (including 16S rRNA) from endogenous bacteria and somatic cells. If the NASBA works, we plan to use toehold biosensors to screen crude milk samples collected from different regions and compare the results with other methods, such as LAMP and PCR (Barboza-Corona et al., 2026), CRISPR/Cas cell-free biosensors, BERA, and electrochemical DNA biosensors, as well as with traditional microbiological assays (De Puig Guixe et al., 2025; Hadjilouka et al., 2020; Saini et al., 2020).

## 5 Conclusion

We developed a toehold-switch biosensor for detecting *L. monocytogenes* in raw milk. Toeholds were activated by synthetic triggers based on the V2 hypervariable region of the 16S rRNA gene and by *Listeria* RNA from pure cultures or from milk artificially contaminated with the bacterium. To our knowledge, no previous study has shown that a toehold switch can be directly activated with bacterial RNA extracted from *L. monocytogenes* pure cultures or from raw milk contaminated with the bacterium. This work demonstrates the utility of the toehold biosensor for detecting a pathogenic bacterium in a food matrix, thereby extending the application of toehold switches beyond viruses and the human microbiota to bacteria in complex food matrices such as raw milk. Because the fold change in bacterial pure culture or in crude milk contaminated with *L. monocytogenes* is low, indicating a weak signal, it will be important to consider enriching the trigger region using a protocol such as NASBA to increase sensitivity. Although results show that Toehold B is activated by *Listeria* RNA in contaminated raw milk, this does not yet constitute full validation for food testing. Finally, the present study laid the foundation for future validation, which should include the determination of the limit of detection for *L. monocytogenes* (CFU/mL), use of foodborne pathogens in raw milk as controls (e.g., *Salmonella* spp., *Staphylococcus aureus*), and additional testing of raw milk samples before the method can be used for on-site detection and risk assessment.

## Data Availability

The raw data supporting the conclusions of this article will be made available by the authors, without undue reservation.

## Footnotes

1
https://www.ncbi.nlm.nih.gov/

2
https://www.idtdna.com/page
