# Supplementary material for: Synthetic toehold biosensors for detecting Listeria monocytogenes in raw milk
Source: Front Microbiol. 2026 Jul 24;17:1891217. doi: 10.3389/fmicb.2026.1891217 (PMC13447367; doi:10.3389/fmicb.2026.1891217)
Supplement: Supplementary file 1 [file Data_Sheet_1.pdf]

Synthetic toehold biosensors for detecting *Listeria monocytogenes* in raw milk

Víctor M. Carballo-Uicab[1#], Luz E. Casados-Vázquez[1,2,3], Drew Endy[4*], José E. Barboza-Corona[1,2*#]

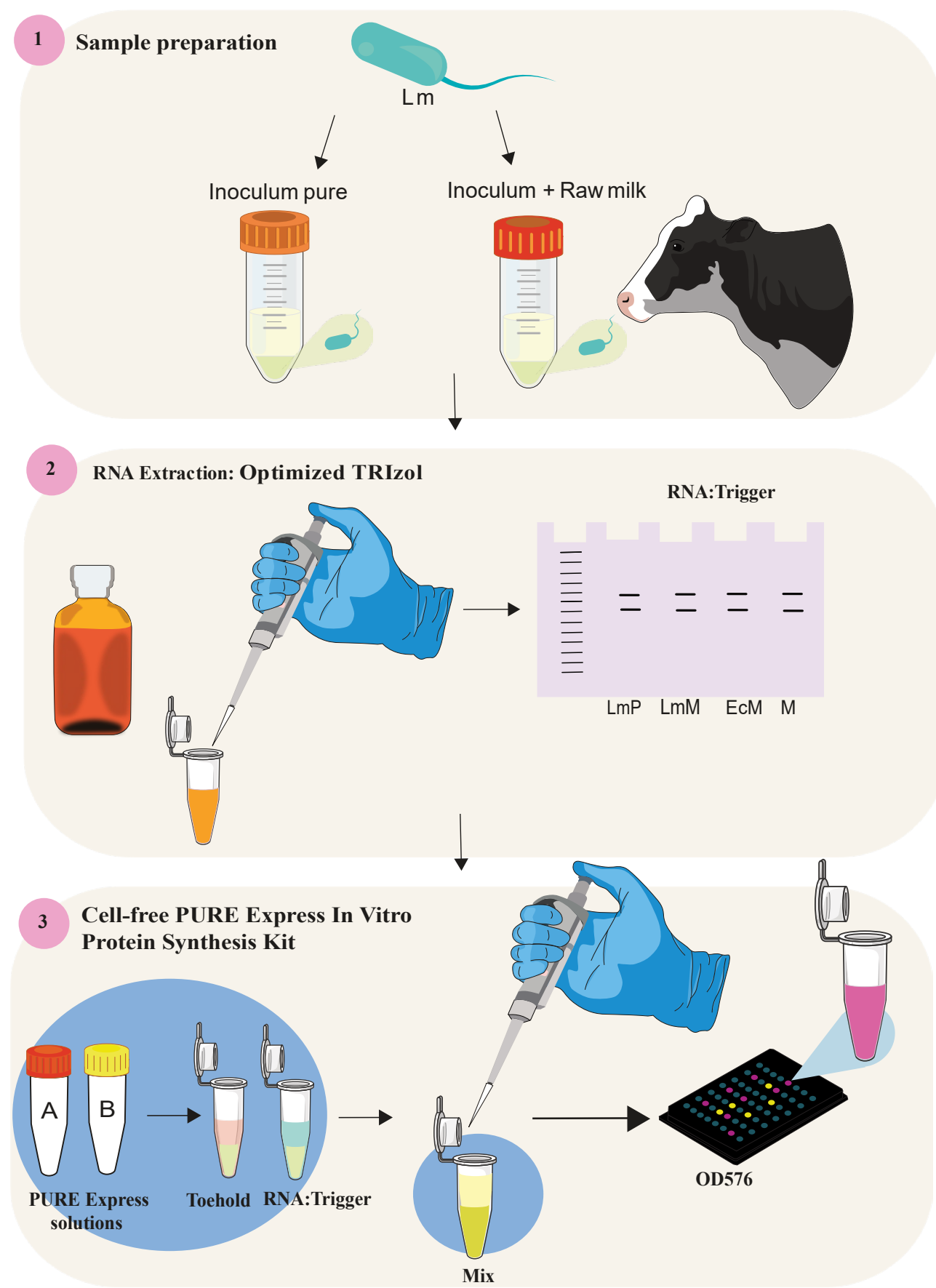

Figure S1. Schematic representation of the experimental workflow for *Listeria monocytogenes* detection using toehold biosensors in a cell-free system. The process consists of three main stages: (1) Sample preparation: Inoculating *L. monocytogenes* (Lm) into pure culture media and a raw milk matrix. (2) RNA extraction: Isolating total RNA using an optimized TRIzol-based protocol. The conceptual gel electrophoresis shows the verification of extracts from *L. monocytogenes* in pure culture (LmP) and milk (LmM), as well as *E. coli* negative control and milk (EcM) and Milk (M). The extracted RNA acts as the "RNA:Trigger" for the biosensor. (3) Cell-free PURExpress assay: Assembling the *in vitro* protein synthesis reaction by combining PURExpress solutions (A and B), the toehold switch biosensor, and the extracted RNA trigger. The reaction mixture is incubated, and the color change is measured by spectrophotometry at 576 nm (OD576). (Figure created with Affinity Designer 2.6.4).

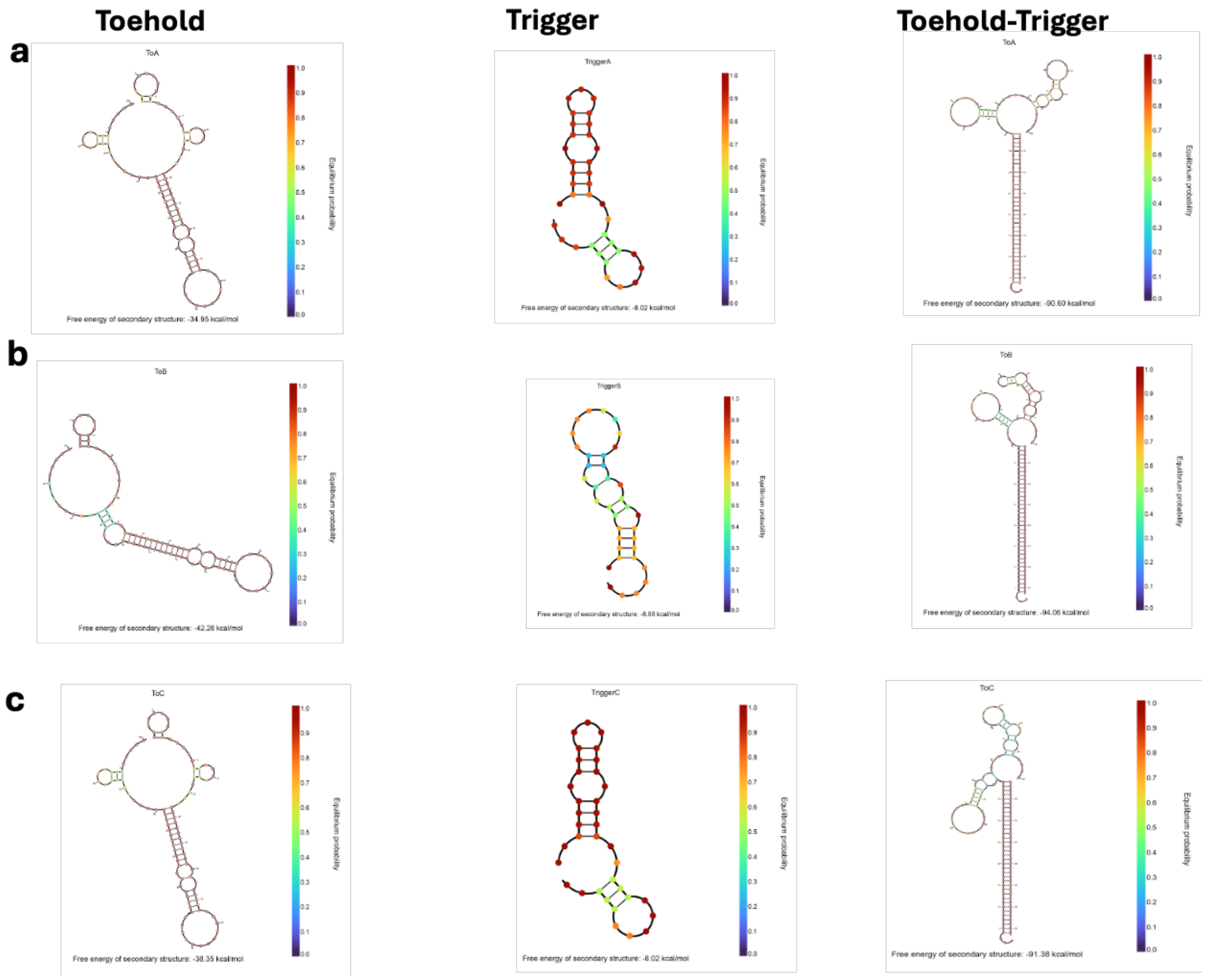

Figure S2. Secondary structure of the toehold switches, triggers, and their interactions. (a) Toehold switch A, (b) Toehold switch B, and (c) Toehold switch C. On the left, from top to bottom, are the toehold switches A, B, and C. In the middle are the triggers A, B, and C. On the right is the interaction between the corresponding toehold and trigger (toehold A-trigger A, toehold B-trigger B, and toehold C-trigger C). Structures and interactions were obtained with the Nucleic Acid Package (NUPACK) (Zadeh et al., 2011).

Table S1. Toehold switches and triggers sequences and other important sequences

**Name**	**Toehold sensor sequences**	**Trigger sequences**
Toehold A	gggacccuaccaacuagcuaaugcaccgcgggcccaucuggacuuuagaacagaggagauaaagaugagaugggcccgaaaccuggcggcagcgcaaaag	agaugggcccgcggugcauuagcuaguugguagggu
Toehold B	gggccauuacccuaccaacuagcuaaugcaccgcgggccggacuuuagaacagaggagauaaagaugggcccgcggugcaaccuggcggcagcgcaaaag	ggcccgcggugcauuagcuaguugguaggguaaugg
Toehold C	gggcccuaccaacuagcuaaugcaccgcgggcccaucugggacuuuagaacagaggagauaaagaugcagaugggcccgaaccuggcggcagcgcaaaag	cagaugggcccgcggugcauuagcuaguugguaggg
Toehold E. coli	gaacgtcaatgagcaaaggtattaactttactcccttggactttagaacagaggagataaagatgaagggagtaaagaaccuggcggcagcgcaaaag	aagggagtaaagttaatacctttgctcattgacgtt
T7 promoter	taatacgactcactatagg	T7 promoter sequence
T7 terminator	tagcataaccccttggggcctctaaacgggtcttgaggggttttttg	T7 transcriptional terminator sequence
Linker	aacctggcggcagcgcaaaag	Linker used to connect the toehold switch with *lacZ*

Table S2. Thermodynamic parameters of toeholds A, B, and C*

Toehold switch	Toehold ΔG MFE# (kcal/mol)	Trigger ΔG MFE# (kcal/mol)	Toehold-Trigger ΔG MFE # (kcal/mol)	Interpretation
A	- 34.95	- 8.02	- 90.60	Moderate stability
B	- 42.28	- 6.88	- 94.06	Most stable
C	- 38.35	- 8.02	- 91.38	Intermediate stability

*Data were obtained using the Nucleic Acid Package (NUPACK) (Zadeh et al., 2011).
MFE ΔG, minimum free energy of Gibbs.

Table S3. Primers used to construct the Toehold/pSNI and Trigger/pSNI recombinant plasmids and for other assays.

**Name**	**Sequence (5´-3´)**	**Description**
Oligo 1Fw	atgaccatgattacggattcac	Forward primer to amplify *lacZ*
Oligo 2Rv	atgcctgaggtttcagcaa	Reverse primer to amplify *lacZ*
Oligo 3Fw	gctctagagagcgctaatacgactcactataggg	Amplified a short fragment of pSNI and the T7 promoter
Oligo 4Rv	caaaaaacccctcaagacccg	Reverse T7 terminator
Oligo 5Fw	tagcataaccccttggggcctctaaacgggtcttgaggggttttttg	Amplified the positive strand of the T7 terminator
Oligo 6Rv	tatagtgagtcgtattagcgctctctagagc	Amplified the negative strand of T7 promoter and a short sequence of the pSNI
Oligo 7Rv	gaatccgtaatcatggtcatc	Amplified the negative strand of a short fragment of the 5´ of *lacZ*
Oligo 8Fw	gaaatctgaagctctagagagcgctaatacg	Contain 24 nt of the pSNI vector and 7 nt of the positive strand of the T7 promoter
LimFw<br><br>LimRv	gcttaataacccctgaccg<br><br>aatcccaatcttcctaaccac	Specific to amplify a transcriptional regulator of the *L. monocytogenes* MeR family.

Table S4. Statistical analysis of the fold-change[#] obtained with different samples

**Group[##]**	**Fold Change**
LmP	1.806318 ± 0.007793[a]
LmM	1.527847 ± 0.025906[b]
EcM	0.916075 ± 0.002305[c]
M	0.738237 ± 0.015857[d]
C	0.157288 ± 0.001546[e]

[#]Fold change was calculated by dividing the absorbance of the toehold activated with trigger by that of the toehold alone

[##] Different RNA samples were assayed with the Toehold B biosensor. LmP,RNA from a pure culture of *L. monocytogene*s; LmM, raw milk inoculated with *L. monocytogenes; EcM,* raw milk inoculated with *E. coli*; M, RNA from crude milk; C, a mix reaction without toehold and trigger.

Different letters in the same column (a, b, c, d, e) indicate significant differences at $p < 0.05$, determined by ANOVA with Tukey's multiple-comparison test.
